# Editorial: Metabolic reprogramming in breast cancer

**DOI:** 10.3389/fonc.2022.1081171

**Published:** 2022-11-10

**Authors:** Cristian Scatena, Antonio Giuseppe Naccarato, Bela Ozsvari, Domenica Scumaci

**Affiliations:** ^1^ Division of Pathology, Department of Translational Research and New Technologies in Medicine and Surgery, University of Pisa, Pisa, Italy; ^2^ Department of Laboratory Medicine, Pisa University Hospital, Anatomia Patologica 1 Universitaria, Pisa, Italy; ^3^ Translational Medicine, School of Science, Engineering and the Environment (SEE), University of Salford, Greater Manchester, United Kingdom; ^4^ Research Center on Advanced Biochemistry and Molecular Biology, Department of Experimental and Clinical Medicine, Magna Græcia University of Catanzaro, Catanzaro, Italy

**Keywords:** breast cancer, metabolic reprogramming, metabolic therapy, breast cancer treatment, resistance mechanisms

Metabolic reprogramming is an emerging hallmark of breast cancer. A common characteristic of tumor cells is the ability to obtain nutrients from a nutrient-deprived environment and to use them to sustain cancer progression, within crucial metabolic pathways including altered metabolism of glucose, lipids, and amino acids. Also, altered metabolism has been recognized as one of the major mechanisms of resistance to therapies. Important advances have been made to elucidate key mechanisms of metabolic reprogramming which will make possible novel strategies for overcoming breast cancer. However, for metabolic therapy to be effective there is a need to clearly understand the metabolic underpinnings of the different subtypes of breast cancer as well as the role the standard-of-care therapies play in targeting the metabolic phenotype.

This Research topic consists of three original research articles and three reviews which focus on the major metabolically adapted pathways in breast cancer exploring either potential metabolic targets and corresponding agents for breast cancer treatment or how metabolic adaption influences breast cancer progression and resistance to therapies.

## Glucose and fatty acid metabolism

In 1956 Otto Warburg first observed that aerobic glycolysis is a hallmark of glucose metabolism in cancer. As reported by Kang et al. cancer cell–derived extracellular vesicles (EVs) can modulate glucose metabolism to induce cell proliferation and an aggressive cancer phenotype. In details, the authors performed proteomic and transcriptomic analyses to investigate the changes in gene expression profiles in the recipient MCF7 cell line and MDA-MB-231 cell-derived EVs. Interestingly, they observed that the uptake of glucose analog [^18^F]fluorodeoxyglucose by the recipient MCF7 cells significantly increases after coculture with MDA-MB-231 cells. Moreover, MCF7 cells increase the capacity to proliferate and dedifferentiate. These data suggest that cancer cells can induce a transition to an aggressive phenotype to activate glucose metabolism *via* EVs. This interesting study may serve as a cornerstone for future research on interactions between cancer cells. The increased uptake of glucose and hyperactivated glycolysis, together with the accumulation of lactate, represent the main alterations in glucose metabolism associated with cancer. Several studies also suggest that aberrant lipid metabolism plays an important role in the adaptation of cancer cells to cellular stress induced by treatments. Tokes et al. in their review discussed extensively the changes in glucose and fatty acid pathways induced by routinely applied chemotherapeutic drugs used in neoadjuvant or adjuvant settings of breast cancer. The authors also analyzed the underlying molecular mechanisms together with novel therapeutic strategies.

## Hypoxia and tumor microenvironment

Solid tumors commonly present regions of hypoxia which increases oxidative stress through the production of mitochondrial reactive oxygen species (ROS) and drives changes in gene expression increasing the risk of cancer metastasis. To improve survival, tumor cells respond by activating genes that modify cell metabolism. In the study by Kiesel et al. hypoxia induces a greater suppression of glutamine to glutamate conversion in murine mammary cancer cell lines (13% in metastatic compared to 7% in nonmetastatic cells). The Authors also suggest that hypoxia increases expression of genes involved in antioxidant response in metastatic compared to nonmetastatic cells, including glutamate cysteine ligase catalytic and modifier subunits and malic enzyme 1. Interestingly, hypoxia also increases the transcriptional (4.2-fold) and translational (6.5-fold) expression of activating transcription factor 4 (ATF4) the main effector of the integrated stress response (ISR) pathway. These findings indicate that metastatic cells utilize hypoxia for metabolic reprogramming and induction of antioxidant defense for survival. In the last decade, therapeutic opportunities for targeting vulnerabilities in tumor cells and the immune microenvironment (TIME) are emerging as a key area of research. The review by Kalyanaraman et al. focused on hypoxia, tumor acidity, the bidirectional proton-coupled monocarboxylate transporters (MCTs) of lactate, mitochondrial oxidative phosphorylation (OXPHOS), and redox enzymes in the tricarboxylic acid cycle as vulnerable targets for cancer therapy. In particular, mitochondria-targeted small molecule inhibitors of OXPHOS inhibit tumor proliferation and growth whereas MCT inhibitors exert synthetic lethality in combination with metformin in cancer cells. Recently, the TIME was proposed as a vulnerable therapeutic target: an acidic milieu is one of the hallmarks of cancer and approaches to reverse the acidification of the TIME could facilitate chemotherapy and immunotherapy.

## Metabolic reprogramming in breast cancer diagnosis and drug resistance

New technologies with high sensitivity and specificity for early diagnosis and monitoring of breast cancer are emerging, as automatic breast full volume scanning system (ABVS). Within this contest, metabolic reprogramming may provide potential biomarkers to combine with ABVS with the aim to improve BC diagnosis accuracy. Intriguingly, in serum samples from 70 patients Liu et al. identified 54 different metabolites that discriminate between benign and malignant breast tumors and 17 different metabolites between invasive and non-invasive breast cancer; notably, differential metabolite analysis was able to reduce the missed diagnosis rate of ABVS. Metabolic reprogramming is gaining much attention not only for early diagnosis but also for monitoring drug resistance in breast cancer. In their review, Lv et al. elucidated the relationship between dysregulation of cellular metabolism and resistance to treatment in breast cancer with particular attention to the regulation of glucose, amino acid and fatty acid metabolism, autophagy and OXPHOS, offering promise for novel targeted therapies.

## Author contributions

All authors listed have made a substantial, direct, and intellectual contribution to the work, and approved it for publication.

## Acknowledgments

We thank the authors of the articles published as a part of this Research Topic for their valuable contributions.

## Conflict of interest

The authors declare that the research was conducted in the absence of any commercial or financial relationships that could be construed as a potential conflict of interest.

## Publisher’s note

All claims expressed in this article are solely those of the authors and do not necessarily represent those of their affiliated organizations, or those of the publisher, the editors and the reviewers. Any product that may be evaluated in this article, or claim that may be made by its manufacturer, is not guaranteed or endorsed by the publisher.

